# Association between molar–incisor hypomineralization and developmental dental anomalies in U.S. children: a cross-sectional study

**DOI:** 10.1007/s00784-026-07239-2

**Published:** 2026-11-05

**Authors:** Azza Tagelsir Ahmed, Juno Park, Evelina Kratunova, Majd Alsaleh, Sahar Alrayyes, Yousef Khader, Michielle Kagei, Helen Rodd, Susej Bandres Rivas, Hani Nazzal

**Affiliations:** 1 Department of Pediatric Dentistry, University of Illinois Chicago College of Dentistry, Chicago, IL USA; 2 Department of Public Health, Jordan University of Science and Technology, Irbid, Jordan; 3 Unit of Oral Health, Dentistry and Society, School of Clinical Dentistry, University of Sheffield, Sheffield, S10 2TA UK; 4 Department of Clinical Oral Health Sciences, College of Dental Medicine, Qatar University, Doha, Qatar

**Keywords:** Molar incisor hypomineralization, Developmental dental anomalies, Short root anomaly, Taurodontism, Dental radiography, Pediatric dentistry, Cross sectional study, Children

## Abstract

**Objectives:**

To compare the frequency of developmental dental anomalies (DDAs) in children with and without molar–incisor hypomineralization (MIH), and to evaluate whether any association is independent of demographic factors.

**Materials and methods:**

This comparative cross-sectional study included children aged 6–14 years with and without MIH. Demographic data were collected by structured maternal questionnaire. MIH was diagnosed by calibrated examiners using a validated MIH Index. DDAs were assessed on panoramic radiographs by a blinded examiner using predefined criteria. Group comparisons used Fisher’s exact test (two-sided), and multivariable logistic regression estimated adjusted odds ratios (aORs) with 95% confidence intervals (CIs), adjusting for country of birth, sex, and ethnicity.

**Results:**

In total, 159 children were enrolled. Any DDA was more frequent in the MIH group than in the non-MIH group (​39.5% vs. 13.7%; *p* < 0.001​). Short root anomaly (14.0% vs. 1.4%; *p* = 0.003) and taurodontism (7.4% vs. 0.0%; *p* = 0.031) were also more frequent with MIH, whereas microdontia of the maxillary lateral incisors was more frequent numerically but not significantly (5.8% vs. 0.0%; *p* = 0.063). MIH remained independently associated with any DDA in multivariable analysis (aOR = 4.52; 95% CI: 1.97–10.33; *p* < 0.001).

**Conclusions:**

Children with MIH had greater odds of concurrent DDAs than children without MIH, most often short root anomaly. Individual anomaly estimates were imprecise owing to few events.

**Clinical relevance:**

Children with MIH may present with concurrent DDAs, highlighting the potential value of comprehensive clinical assessment and appropriate radiographic evaluation when clinically indicated.

## Introduction

Molar–incisor hypomineralization (MIH) is a qualitative developmental enamel defect affecting one or more first permanent molars, frequently in association with permanent incisors [1, 2]. It is characterized by demarcated opacities of variable color and severity and, in more severe cases, post-eruptive enamel breakdown, atypical restorations or caries, and early tooth loss [1, 2]. Clinically, MIH is associated with hypersensitivity, rapid enamel disintegration, increased caries susceptibility, and treatment challenges, and may adversely affect oral health-related quality of life while imposing psychosocial and economic burdens on affected children and their families [1–4].

MIH is among the most common developmental defects of enamel, with recent global prevalence estimates of 13.5%–15.5% [5, 6]. Marked geographic variation has been reported, likely reflecting interactions among environmental, biological, and genetic determinants [6, 7]. Although prevalence appears relatively high in North America, U.S. data remain limited [6, 8].

Developmental dental anomalies (DDAs) encompass disturbances in tooth number, size, morphology, root development, and eruption [9]. Relevant findings include hypodontia and supernumerary teeth, microdontia and structural morphological variations such as dens invaginatus or evaginatus, root-related abnormalities including taurodontism and short root anomaly, and disturbances of eruption or tooth position [9]. DDAs may occur in isolation, although the co-occurrence of certain phenotypes within individuals and families has been documented, supporting the possibility of partially overlapping developmental and genetic susceptibility [9, 10]. Developmental disturbances affecting enamel formation and odontogenesis may involve genetic, epigenetic, and environmental influences [11]. Developmental enamel defects have also been reported together with abnormalities of tooth number and size in children with systemic or developmental conditions [12, 13]. However, individual DDAs may arise at different stages of odontogenesis and through distinct etiologic pathways, so co-occurrence does not by itself imply a shared mechanism [9].

Similar patterns of co-occurrence have prompted investigation of DDAs in children with MIH. Previous studies have evaluated anomalies of tooth number, size, morphology, and eruption in this population [14–17]. Higher frequencies of selected DDAs have been reported in some MIH cohorts [14–16]. In a recent international study, hypodontia, microdontia of the maxillary lateral incisors, dens invaginatus, and dens evaginatus were more frequent among children with MIH, with geographic variation in the distribution of these findings [15]. Other investigations have not demonstrated significant associations between MIH and selected developmental abnormalities, including dental agenesis [17]. These observations provide a rationale for evaluating a broad spectrum of clinically and radiographically identifiable developmental dental findings rather than focusing on a single anomaly.

From a clinical perspective, the co-occurrence of DDAs in children with MIH may further complicate diagnosis, monitoring, treatment planning, and long-term management. This is particularly relevant in pediatric patients for whom treatment decisions may already be complicated by hypersensitivity, post-eruptive enamel breakdown, and uncertainty regarding the prognosis of affected first permanent molars. The presence of coexisting DDAs may therefore expand the burden of MIH beyond enamel management alone and strengthen the need for early comprehensive assessment and interdisciplinary treatment planning involving pediatric dentists and orthodontists [18].

Dental phenotype is influenced by ancestry, and several studies have shown that some DDAs are more prevalent in specific population groups [19, 20]. In contrast, evidence regarding disparities in enamel defects, including MIH, across ethnic groups remains inconsistent [8, 21, 22]. This issue is particularly relevant in the United States, where pediatric populations are highly diverse and region-specific data on MIH and its coexisting dental findings remain limited. Despite growing international interest in the relationship between MIH and DDAs, few studies have examined this association in children in the United States, and data from diverse cohorts remain especially scarce.

Given these gaps, and the limited evidence from ethnically diverse pediatric populations in the United States, further investigation is needed. The aims of the present study were to compare the frequency of DDAs in children with and without MIH, to determine whether MIH was independently associated with the presence of DDAs after adjustment for demographic factors, and to assess the distribution of DDAs according to sex, ethnicity, and country of birth.

## Materials and methods

This comparative cross-sectional study was approved by the Institutional Review Board of the University of Illinois Chicago (STUDY #2023 − 1293). Written informed consent was obtained from parents or legal guardians before enrollment, and assent was obtained from all participating children. Participants were recruited between October 2024 and December 2025 from the Department of Pediatric Dentistry at the University of Illinois Chicago. Eligible participants were identified from the pool of current and new patients attending the clinic during the study period, and initial screening and recruitment were performed by a research team member. The study used a clinic-based convenience sample of eligible children drawn from the same clinical source population, from which participants with and without MIH were recruited.

Children diagnosed clinically with MIH comprised the MIH group, whereas children without clinical evidence of MIH comprised the non-MIH comparison group. Selection of participants was based on MIH status and the predefined eligibility criteria only. The presence or absence of DDAs was not considered during recruitment or group assignment. Eligibility criteria for both groups were age 6–14 years, American Society of Anesthesiologists (ASA) physical status I or II, attendance accompanied by the biological mother, ability to tolerate a comprehensive clinical examination, and availability of a diagnostic panoramic radiograph in the electronic health record. ASA I refers to a normal healthy child, whereas ASA II refers to a child with mild systemic disease without substantive functional limitation. Children with craniofacial or syndromic conditions, those undergoing genetic testing for craniofacial abnormalities, those with fixed orthodontic appliances, and those with behavioral limitations precluding a comprehensive examination were excluded. Attendance with the biological mother was required because the study questionnaire was completed by the mother and was specifically designed to capture prenatal, perinatal, and postnatal history relevant to the child. No a priori sample size calculation was performed and the study sample consisted of all eligible participants recruited during the study period.

Clinical examinations were performed with the child seated in a dental chair under standard operatory illumination. Teeth were cleaned as needed to remove visible plaque or debris and were examined after gentle air drying. MIH was assessed clinically during the routine dental visit by one of four trained and calibrated examiners using the validated MIH Index described by Ghanim et al., (2017) [23]. A child was classified as affected by MIH when at least one first permanent molar showed a demarcated opacity, post-eruptive enamel breakdown, an atypical restoration or caries attributable to MIH, or extraction due to MIH, with or without involvement of the permanent incisors. MIH severity was classified at the participant level according to the highest severity observed in at least one first permanent molar. Mild MIH was defined as the presence of creamy-white or yellow-brown demarcated opacities without post-eruptive enamel breakdown. Moderate/severe MIH was defined as the presence of post-eruptive enamel breakdown, atypical restoration, atypical caries, or a tooth missing due to MIH, according to the criteria described by Ghanim et al. [23]. The MIH Index was selected because it provides a standardized method for distinguishing MIH from other developmental enamel defects and for recording both lesion severity and lesion extent in a reproducible manner. Under this system, teeth are classified as sound, affected by non-MIH enamel defects, showing demarcated opacities, post-eruptive enamel breakdown, atypical restorations, atypical caries, extraction due to MIH, or not scorable. Demarcated opacities are further characterized by color, including white/creamy and yellow/brown presentations, and lesion extent is recorded according to the proportion of the affected surface. Examiner training and calibration were based on the validated MIH Index and its associated training materials. Examiners were trained to identify MIH, distinguish it from other developmental enamel defects, and score lesion severity and lesion extent consistently. The calibration materials included a structured charting form and a training deck containing 20 clinical cases scored at the buccal/labial, lingual/palatal, and occlusal/incisal surfaces for both lesion score and lesion extent across repeated calibration sessions. Inter-examiner agreement was assessed by comparing each examiner’s scores with those of the benchmark examiner using Cohen’s kappa coefficient, and intra-examiner agreement was assessed by repeated scoring of the calibration cases across sessions. Inter-examiner kappa values ranged from 0.90 to 1.00 for lesion score and from 0.71 to 0.93 for lesion extent.

Demographic and background data were collected using a structured questionnaire specifically developed for the purposes of this study and implemented in REDCap^®^ (Research Electronic Data Capture; Vanderbilt University, Nashville, TN, USA), a secure web-based electronic data capture platform hosted by the University of Illinois Chicago Center for Clinical and Translational Science. The questionnaire was completed by the participant’s biological mother on a secure iPad during the child’s dental visit. The instrument was designed to obtain information on demographic characteristics and on prenatal, perinatal, and postnatal factors potentially relevant to the child’s dental development. These domains included maternal health before and during pregnancy, pregnancy medications and complications, delivery and birth-related factors, infant feeding practices, environmental exposures during early childhood, and the child’s medical history during the first 2 years of life. Variables collected for the present analysis included age, sex, country and state of birth, country and state of residence during the first 2 years of life, and parental education.

DDAs were assessed on existing panoramic radiographs of diagnostic quality by a single examiner who was blinded to MIH status. The radiographs were accessed through the institutional axiUm electronic health record system (axiUm version 7.11.03.11675; Exan Software, Las Vegas, NV, USA) and viewed using DEXIS imaging software (DEXIS/Dental Imaging Technologies Corporation, Quakertown, PA, USA). Panoramic radiographs were acquired using a DEXIS™ ORTHOPANTOMOGRAPH™ OP 3D™ unit (DEXIS/Dental Imaging Technologies Corporation, Quakertown, PA, USA). Exposure parameters were selected according to the manufacturer’s age- and patient-size–appropriate panoramic protocols. Radiographic assessment was performed after completion of the clinical examination and followed the standardized protocol described by Rodd et al., (2023) [24], which was developed for the international investigation of MIH and dental anomalies. Under this protocol, anomalies of tooth number, size, form, and eruption were systematically evaluated on panoramic radiographs. Hypodontia was defined as congenital absence of a permanent tooth germ, excluding third molars. Supernumerary teeth were recorded when an additional tooth or tooth germ was present beyond the normal complement of the permanent dentition. Microdontia was assessed in the permanent maxillary lateral incisors and recorded when the tooth appeared distinctly reduced in mesiodistal width or peg-shaped relative to the expected morphology. Dens invaginatus and dens evaginatus were recorded when characteristic radiographic evidence of enamel-dentin infolding or an accessory tubercle was present. Odontoma was recorded when a well-defined radiopaque lesion containing tooth-like structures or a conglomerate radiopaque mass consistent with a compound or complex odontoma was identified radiographically. Ectopic maxillary canines were recorded radiographically when the permanent maxillary canine showed an abnormal eruption path or position. Ectopic eruption of the first permanent molars was recorded radiographically when an abnormal eruption path was associated with distal resorption of the second primary molar, including cases in which the first permanent molar had subsequently erupted. These findings were recorded based on their radiographic presentation and were not intended to imply a specific etiology. Taurodontism was assessed only in participants with complete apical development of the first permanent mandibular molars, using digital vertical measurements on panoramic radiographs according to the method of Lai and Seow (1989) [25]. The crown-body to root ratio was calculated, and teeth were classified as normal (cynodont) when the ratio was < 1.10, hypotaurodont when 1.10–1.29, mesotaurodont when 1.30–2.00, and hypertaurodont when > 2.00. Short root anomaly (SRA) was assessed in the six anterior teeth and all premolars according to the criteria described by Lind (1972) [26]. A root-to-crown ratio greater than 1:1 was considered normal, whereas a ratio equal to or less than 1:1 was considered diagnostic of SRA. SRA was assessed only when root development was sufficiently advanced to permit reliable crown-to-root measurement. Images with substantial distortion, positioning error, or projection artifact that precluded reliable measurement were not used for SRA classification. The primary outcome variable for the present study was the presence of any DDA.

Descriptive statistics were calculated as means, frequencies, and percentages. For categorical variables, differences in the distribution of participants between the MIH and non-MIH groups were evaluated using Fisher’s exact test (two-sided), with statistical significance set at α = 0.05. Fisher’s exact test was applied to all comparisons because expected cell counts were below 5 for several of the less frequent anomalies. Multivariable logistic regression was used to evaluate the independent association between MIH and the presence of any DDA after adjustment for sex, ethnicity, and country of birth. Statistical analyses were performed using IBM SPSS Statistics, version 24.0 (IBM Corp., Armonk, NY, USA). Results are presented as adjusted odds ratios (aORs) with 95% confidence intervals (CIs).

## Results

**Fig. 1:**
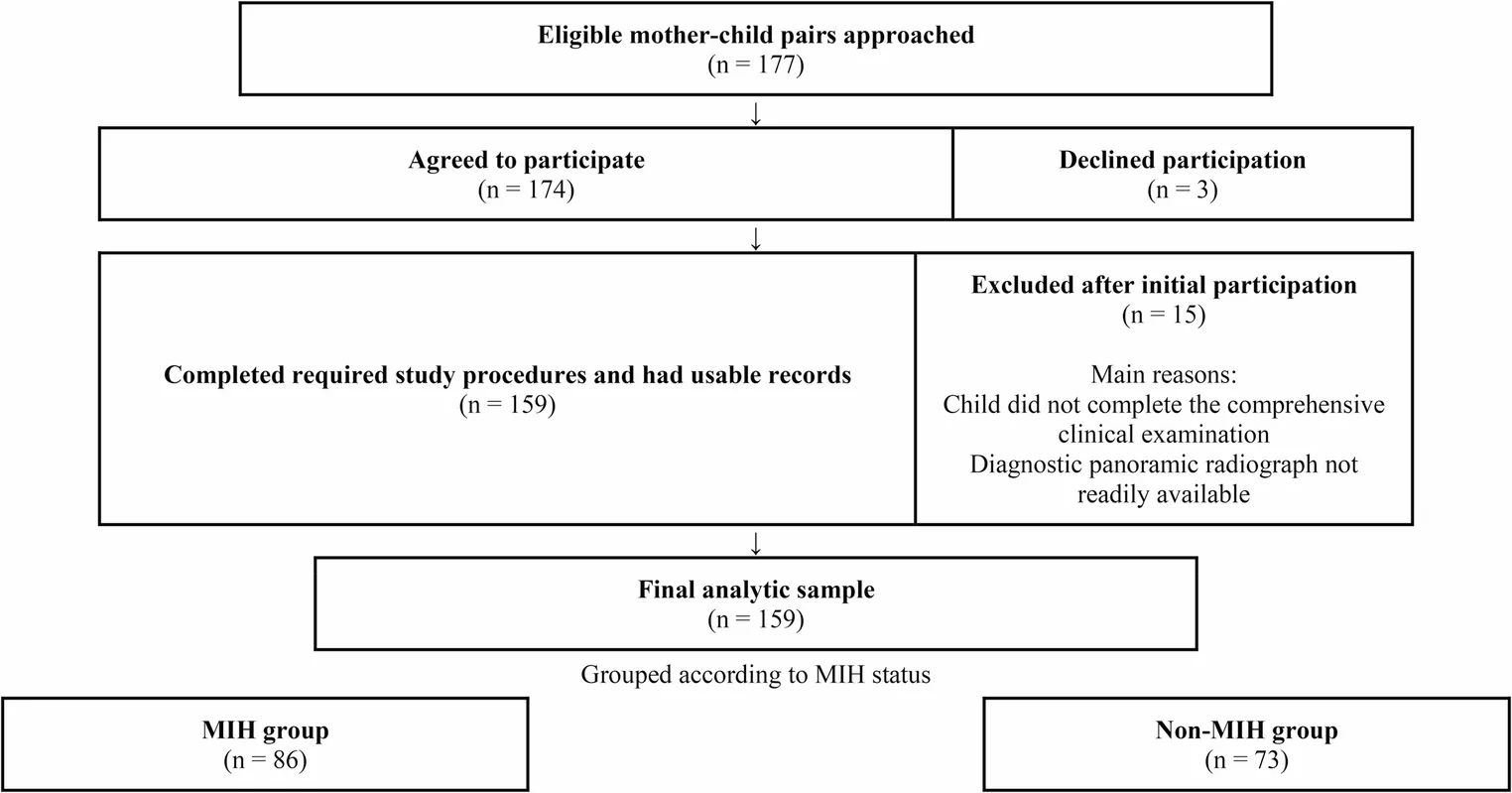
Participant flow diagram. Abbreviation: MIH, molar-incisor hypomineralization

A total of 177 eligible mother–child pairs were approached for participation. Three declined to participate, and 15 were excluded after initial participation, primarily because the child did not complete the comprehensive clinical examination or a diagnostic panoramic radiograph was not readily available. The final analytic sample comprised 159 children, including 86 with MIH and 73 without MIH (Fig. 1), corresponding to an overall inclusion rate of 89.8% among those approached. The mean age was 9.43 ± 1.97 years in the MIH group and 8.54 ± 1.77 years in the non-MIH group. Females accounted for 51.8% of the MIH group and 59.7% of the non-MIH group. Most participants were born in the United States (MIH: 78.6%; non-MIH: 85.7%) and had resided there during the first 2 years of life (MIH: 76.7%; non-MIH: 87.5%). Hispanic children comprised 66.3% of the MIH group and 70.4% of the non-MIH group. Among children born in the United States, 96.8% of those in the MIH group and 93.1% of those in the non-MIH group were born in Illinois. Most mothers had completed high school (MIH: 38.1%; non-MIH: 51.4%), and the majority of participants had public insurance (MIH: 93.8%; non-MIH: 95.8%). No significant between-group differences were observed in sex, birthplace, early-life residence, ethnicity, maternal education, paternal education, or insurance status (Table 1).

**Table 1:** Sociodemographic characteristics of the study population

Variable	MIH (*n* = 86), n (%)	Non-MIH (*n* = 73), n (%)	*p*-value
Sex			0.338
Male	41 (48.2)	29 (40.3)	
Female	44 (51.8)	43 (59.7)	
Birthplace			0.298
United States	66 (78.6)	60 (85.7)	
Other countries	18 (21.4)	10 (14.3)	
Early-life residence during first 2 years			0.123
United States	56 (76.7)	56 (87.5)	
Other countries	17 (23.3)	8 (12.5)	
Ethnicity			0.607
Hispanic	55 (66.3)	50 (70.4)	
Non-Hispanic	28 (33.7)	21 (29.6)	
Mother’s education			0.231
Less than high school	13 (15.5)	5 (6.9)	
High school	32 (38.1)	37 (51.4)	
Some college	27 (32.1)	22 (30.6)	
Graduate/professional	12 (14.3)	8 (11.1)	
Father’s education			0.602
Less than high school	19 (24.1)	12 (18.8)	
High school	38 (48.1)	37 (57.8)	
Some college	17 (21.5)	13 (20.3)	
Graduate/professional	5 (6.3)	2 (3.1)	
Insurance			0.723
Public insurance	76 (93.8)	69 (95.8)	
Private insurance	5 (6.2)	3 (4.2)	

Percentages are based on participants with available data for each variable; denominators therefore vary. Missing responses in the MIH group were: sex, *n* = 1; birthplace, *n* = 2; early-life residence during the first 2 years, *n* = 13; ethnicity, *n* = 3; mother’s education, *n* = 2; father’s education, *n* = 7; and insurance, *n* = 5. No imputation was performed; each variable was analyzed using available cases. All p-values were calculated using two-sided Fisher’s exact tests

Abbreviation: *MIH* molar–incisor hypomineralization

The frequency of any DDA was significantly higher in children with MIH than in children without MIH (39.5% vs. 13.7%; *p* < 0.001). Among the individual anomalies evaluated, short root anomaly was present in 14.0% of children with MIH compared with 1.4% of non-MIH controls (*p* = 0.003), and taurodontism in 7.4% versus 0.0% (*p* = 0.031). Microdontia of the maxillary lateral incisors was present in 5.8% of children with MIH and in no non-MIH controls, but this difference was not statistically significant (*p* = 0.063). The frequency of hypodontia (7.0% vs. 2.7%; *p* = 0.290), supernumerary teeth (1.2% vs. 2.9%; *p* = 0.594), ectopic eruption (7.0% vs. 5.5%; *p* = 0.755), dens invaginatus (1.2% vs. 0.0%; *p* = 1.000), and odontoma (3.5% vs. 0.0%; *p* = 0.250) also did not differ significantly between groups (Table 2).

**Table 2:** Prevalence of DDAs in children with and without MIH

Developmental dental anomaly	MIH (*n* = 86) n (%)	Non-MIH (*n* = 73) n (%)	Total (*n* = 159) n (%)	*p*-value
Hypodontia	6 (7.0)	2 (2.7)	8 (5.0)	0.290
Supernumerary teeth	1 (1.2)	2 (2.7)	3 (1.9)	0.594
Ectopic eruption*	6 (7.0)	4 (5.5)	10 (6.3)	0.755
Microdontia of maxillary lateral incisors	5 (5.8)	0 (0.0)	5 (3.1)	0.063
Dens invaginatus	1 (1.2)	0 (0.0)	1 (0.6)	1.000
Short root anomaly	12 (14.0)	1 (1.4)	13 (8.2)	0.003
Odontoma	3 (3.5)	0 (0.0)	3 (1.9)	0.250
Taurodontism†	6 (7.4)	0 (0.0)	6 (4.0)	0.031
Any DDA	34 (39.5)	10 (13.7)	44 (27.7)	< 0.001

* Ectopic eruption included ectopic eruption of the first permanent molars and ectopic maxillary canines

† Taurodontism was assessed only in participants with complete apical development of the first permanent mandibular molars; percentages for this anomaly are based on eligible participants rather than the full study sample

In stratified analyses, the association between MIH and the presence of any DDA remained significant in males (39.0% vs. 10.3%; *p* = 0.013) and females (40.9% vs. 16.3%; *p* = 0.017). A similar pattern was observed by birthplace: among children born in the United States, 33.3% of those with MIH had at least one DDA compared with 11.7% of non-MIH controls (*p* = 0.005), whereas among children born outside the United States the corresponding proportions were 66.7% and 20.0%, respectively (*p* = 0.046). The largest between-group difference was observed among Hispanic children, among whom 45.5% of those with MIH had at least one DDA compared with 16.0% of non-MIH controls (*p* = 0.001) (Table 3).

**Table 3:** Frequency of developmental dental anomalies in children with and without MIH, stratified by sex, ethnicity, and birthplace

Stratum	Outcome	MIH, *n*/*N* (%)	Non-MIH, *n*/*N* (%)	*p*-value
Sex				
Male	Any DDA	16/41 (39.0)	3/29 (10.3)	0.013
	Short root anomaly	3/41 (7.3)	0/29 (0.0)	0.261
	Taurodontism	3/38 (7.9)	0/28 (0.0)	0.256
Female	Any DDA	18/44 (40.9)	7/43 (16.3)	0.017
	Short root anomaly	9/44 (20.5)	1/43 (2.3)	0.015
	Taurodontism	3/43 (7.0)	0/40 (0.0)	0.242
Ethnicity				
Hispanic	Any DDA	25/55 (45.5)	8/50 (16.0)	0.001
	Short root anomaly	11/55 (20.0)	1/50 (2.0)	0.004
	Taurodontism	4/52 (7.7)	0/47 (0.0)	0.119
Non-Hispanic	Any DDA	8/28 (28.6)	2/21 (9.5)	0.155
	Short root anomaly	1/28 (3.6)	0/21 (0.0)	1.000
	Taurodontism	2/27 (7.4)	0/19 (0.0)	0.504
Birthplace				
Born in the United States	Any DDA	22/66 (33.3)	7/60 (11.7)	0.005
	Short root anomaly	11/66 (16.7)	1/60 (1.7)	0.005
	Taurodontism	3/63 (4.8)	0/59 (0.0)	0.245
Born outside the United States	Any DDA	12/18 (66.7)	2/10 (20.0)	0.046
	Short root anomaly	1/18 (5.6)	0/10 (0.0)	1.000
	Taurodontism	3/17 (17.6)	0/7 (0.0)	0.530

All p-values were calculated using two-sided Fisher’s exact tests

**Fig. 2:**
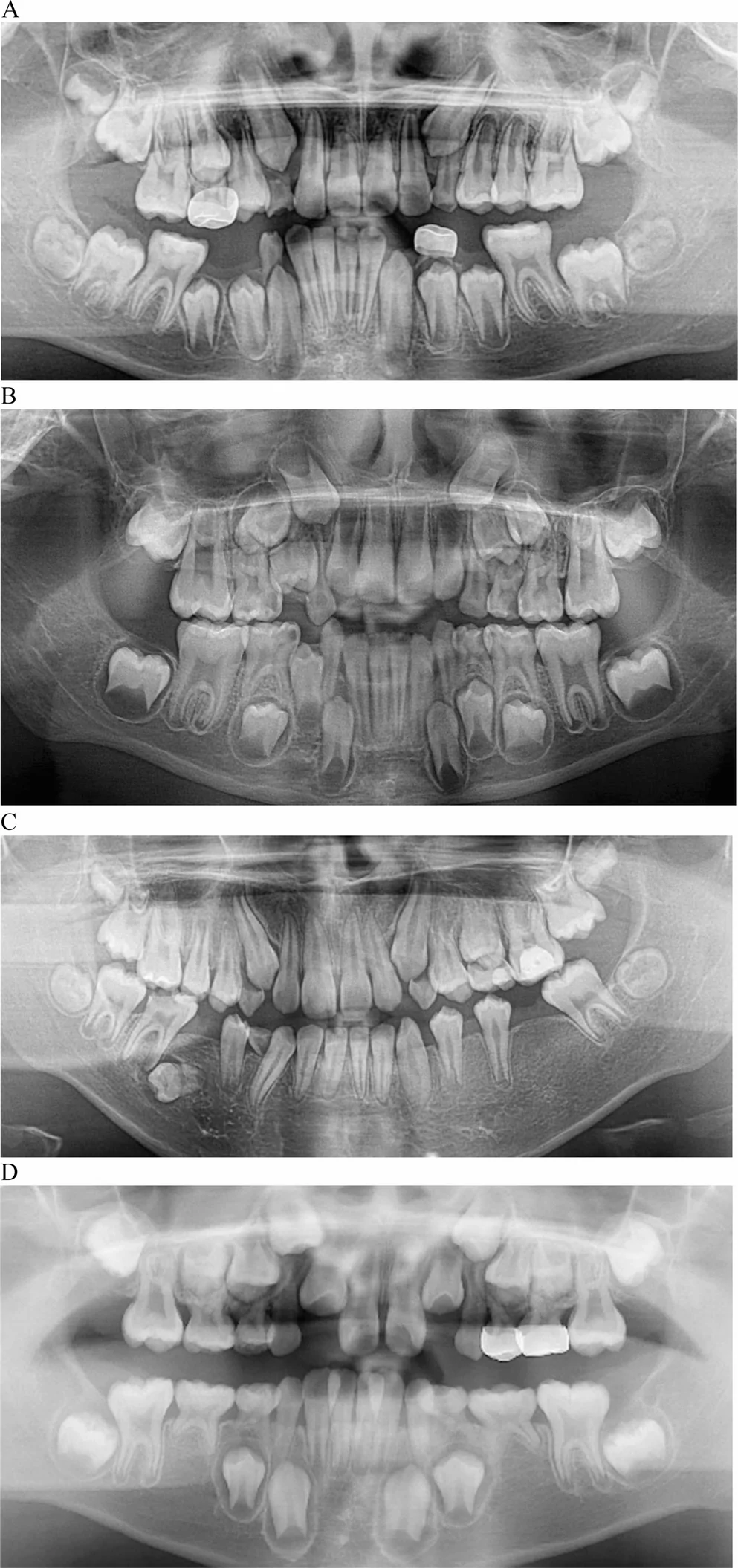
Representative radiographic examples of developmental dental anomalies identified in the study. (**A**) Short root anomaly, affecting the upper central incisors. (**B**) Taurodontism, affecting maxillary first permanent molars. (**C**) Ectopic lower right second premolar. (**D**) Hypodontia of the lower second premolars

Short root anomaly was more frequent in Hispanic children with MIH than in Hispanic controls (20.0% vs. 2.0%; *p* = 0.004), in females with MIH than in female controls (20.5% vs. 2.3%; *p* = 0.015), and in children with MIH born in the United States compared with U.S.-born controls (16.7% vs. 1.7%; *p* = 0.005). Although taurodontism was more frequent in the MIH group overall, stratified analyses by sex, ethnicity, and birthplace did not reach statistical significance. Overall, short root anomaly was identified in 13 children (8.2% of the total study population), 12 of whom were in the MIH group. Most cases were localized; one child showed generalized short root anomaly (Fig. 2).

In multivariable logistic regression, MIH remained independently associated with the presence of any DDA after adjustment for country of birth, sex, and ethnicity (adjusted odds ratio [aOR] = 4.52; 95% confidence interval [CI]: 1.97–10.33; *p* < 0.001). Country of birth (aOR = 1.27; 95% CI: 0.55–2.93; *p* = 0.577), sex (aOR = 1.50; 95% CI: 0.68–3.31; *p* = 0.321), and ethnicity (aOR = 1.90; 95% CI: 0.77–4.71; *p* = 0.165) were not independently associated with the outcome in the adjusted model (Table 4).

**Table 4:** Multivariable logistic regression model for the presence of any DDA

Variable	Adjusted OR (95% CI)	*p*-value
Study group (MIH vs. non-MIH)	4.52 (1.97–10.33)	< 0.001
Country of birth	1.27 (0.55–2.93)	0.577
Sex	1.50 (0.68–3.31)	0.321
Ethnicity (Hispanic vs. non-Hispanic)	1.90 (0.77–4.71)	0.165

When MIH severity was dichotomized at the patient level according to the highest severity score observed in at least one first permanent molar, DDAs were more frequent in children with moderate/severe MIH than in those with mild MIH, although the difference did not reach statistical significance (43.4% vs. 10.0%; *p* = 0.081). Specifically, among the 10 children classified as having mild MIH, 1 had a DDA, whereas among the 76 children classified as having moderate/severe MIH, 33 had a DDA (Table 5).

**Table 5:** Association between MIH severity and the presence of DDAs among children with MIH

MIH severity	Description	No DDA, *n* (%)	Any DDA, *n* (%)	Total, *n* (%)	*p*-value
Mild	Creamy-white or yellow-brown demarcated opacities	9 (90.0)	1 (10.0)	10 (100.0)	0.081
Moderate/severe	Post-eruptive enamel breakdown, atypical restoration, atypical caries, or missing due to MIH	43 (56.6)	33 (43.4)	76 (100.0)	
Total		52 (60.5)	34 (39.5)	86 (100.0)	

## Discussion

The present study found that children with MIH had a significantly higher frequency of DDAs than children without MIH, and that this association remained significant after adjustment for country of birth, sex, and ethnicity. These findings suggest that MIH may co-occur with other developmental dental findings; however, because the anomalies assessed across the dentition may arise at different developmental stages and through different etiologic pathways, the observed association should not be interpreted as evidence of a shared developmental timing or causal mechanism. In this cohort, 39.5% of children with MIH had at least one DDA, compared with 13.7% 14.3% of children without MIH, and MIH was associated with more than fourfold higher adjusted odds of any DDA.

This study is clinically and scientifically relevant because data on the association between MIH and DDAs from the United States remain scarce, particularly in ethnically diverse pediatric populations. With the exception of the California sub-cohort included in the recent international investigation by Nazzal et al., (2025) [15] few U.S.-based data are available. The present cohort therefore contributes important regional data from the U.S. Midwest and expands a literature that has been derived largely from European, Middle Eastern, and South American populations. Differences in demographic composition, environmental exposures, and study design may partly explain why published findings have not been fully consistent [27].

The overall frequency of DDAs overall frequence of DDAs among children with MIH in the present study is broadly comparable to the prevalence reported in the United Kingdom [28], although lower than that reported in Brazilian children with MIH, where more than half of affected children had at least one DDA [14]. At the same time, the adjusted odds ratio observed here was higher than that reported in several previous investigations, in which effect sizes were generally more modest [14, 28]. These differences should be interpreted cautiously. Variation in study design, sample composition, diagnostic criteria, and the range of anomalies assessed may all influence the observed magnitude of association. Overall, the available literature increasingly suggests that MIH is associated with a higher burden of DDAs, even if the exact anomaly profile differs across populations.

The most distinctive finding of the present study was that short root anomaly emerged as the most frequent DDA among children with MIH. To our knowledge, this has not been a prominent finding in previous MIH-DDA studies, which have more commonly emphasized hypodontia, microdontia, or dens anomalies. This observation is noteworthy because short root anomaly has been reported to show familial aggregation, a female predilection, and increased prevalence in Hispanic populations [10, 20, 29]. In the present cohort, more than two-thirds of the study population were Hispanic, and short root anomaly was more frequent among Hispanic children and among females with MIH. The prominence of short root anomaly in this study may therefore partly reflect the demographic profile of the cohort, but it may also indicate that root developmental disturbances deserve greater attention in children with MIH than they have received to date.

This finding also raises biologically plausible questions. A recent study identified a variant in the protocadherin alpha gene cluster as potentially relevant to short root anomaly [30]. Together with reports of familial occurrence of short root anomaly [10, 29] and the higher concordance of MIH among monozygotic twins [31], this supports the possibility that shared genetic susceptibility may contribute to the co-occurrence of MIH and some DDAs. Although the present study cannot address mechanisms directly, it strengthens the rationale for future studies examining whether specific anomalies cluster with MIH because of shared developmental pathways rather than simple coincidence.

The clinical implications of short root anomaly in this setting are important. Orthodontic treatment planning in children with MIH can already be complex because the clinician must determine whether affected first permanent molars are restorable and whether their long-term prognosis justifies extraction [32]. When short root anomaly coexists, this may further complicate decisions regarding orthodontic movement, anchorage, and treatment timing. Reports on the effect of orthodontic treatment in patients with short root anomaly remain limited, and some authors have suggested that severe forms may warrant particular caution [33, 34]. The present findings therefore support careful radiographic evaluation of root morphology in children with MIH, especially when orthodontic treatment is being considered.

In contrast, hypodontia was not significantly associated with MIH in the present study. This finding is consistent with reports from Turkish [35] and Spanish [17] cohorts, but differs from studies that suggested a positive association between MIH and hypodontia [15]. Even within the recent international study, the California sub-cohort showed a relatively low prevalence of hypodontia among children with MIH compared with other regions [15]. The absence of association in the present cohort may therefore reflect population-specific variation rather than a true contradiction of the broader literature. This interpretation is supported by evidence that the prevalence of hypodontia varies geographically and ethnically, with North American populations generally showing lower prevalence than European and Australian populations [36]. The present findings suggest that hypodontia should not be assumed to be a universal marker of MIH-related developmental disturbance.

Taurodontism was also more frequent in children with MIH, and microdontia of the maxillary lateral incisors was observed only in the MIH group, although this difference did not reach statistical significance. The direction of both findings is consistent with previous reports and with the broader concept that disturbances affecting enamel formation may also affect tooth size and root morphology [35, 37]. Taurodontism did not remain significant in stratified analyses, which may reflect limited statistical power in subgroup comparisons and the fact that this anomaly could only be assessed in participants with complete apical development of the first permanent mandibular molars. Given the small number of events for several individual DDAs, the demographic subgroup analyses in Table 3 should be considered exploratory and interpreted cautiously. Even so, the direction of effect remained consistent with the overall pattern of increased developmental anomalies in the MIH group. The ethnicity-related findings should be interpreted with caution. Although the largest difference in the frequency of any DDA was observed among Hispanic children, ethnicity was not independently associated with the outcome in the multivariable model. This suggests that the observed pattern may reflect the anomaly distribution within this cohort rather than an independent effect of ethnicity itself. This is an important distinction. Dental phenotype is influenced by ancestry [38], whereas ethnicity reflects broader social and population-level constructs that may also be linked to environmental exposures and access to care [8, 39]. Future studies should attempt to disentangle these dimensions more explicitly rather than treating them as interchangeable.

The study population was predominantly Hispanic and largely publicly insured, reflecting the sociodemographic characteristics of the university-based clinical setting. Some DDAs, including SRA and taurodontism, have been reported to vary across populations, potentially reflecting differences in genetic background as well as environmental and developmental factors. However, self-reported ethnicity is a sociodemographic construct and should not be considered a direct measure of genetic ancestry. Therefore, the present findings cannot determine whether ancestry-related factors contributed to the observed distribution of DDAs.

Similarly, the absence of a sex-specific interaction in the overall presence of DDAs is consistent with previous reports [15], even though certain individual anomalies, particularly root anomalies, have been reported to show sex predilections [40–45]. The female predominance observed for short root anomaly in this cohort therefore fits the existing literature, but the broader association between MIH and any DDA did not appear to differ meaningfully by sex.

DDAs were numerically more frequent in children with moderate/severe MIH than in those with mild MIH, although this difference was not statistically significant and rests on only 10 children in the mild group. A similar pattern was reported in a recent Brazilian cohort [14]. However, the predominance of moderate/severe MIH in this cohort (76 of 86 children) is substantially higher than would be expected in a population-based epidemiologic sample. This likely reflects the clinic-based convenience sampling strategy and referral characteristics of a tertiary pediatric dental setting, where children with more pronounced symptoms, enamel breakdown, or treatment needs may be more likely to seek or receive care. The severity distribution in this cohort should not be considered representative of the general pediatric population, and the association between MIH severity and DDAs should be interpreted cautiously. It is biologically plausible that more severe MIH reflects a more substantial developmental insult during amelogenesis and that the same insult, or the same underlying susceptibility, could increase the likelihood of additional developmental anomalies. Prior studies have shown that the number of affected molar surfaces is associated with more severe forms of MIH, which supports the idea that severity may act as a proxy for the extent of developmental disruption [8, 45]. Given the small mild-MIH subgroup and the absence of a statistically significant difference, the present data cannot establish whether MIH severity is informative for identifying children with coexisting anomalies, and this question warrants investigation in larger samples with a more balanced severity distribution.

The study has several strengths. MIH and non-MIH participants were recruited from the same clinical source population, MIH was diagnosed using a validated index by trained and calibrated examiners, and DDAs were assessed radiographically by an examiner blinded to MIH status using predefined diagnostic criteria. In addition, the study provides data from a diverse pediatric population in the United States, which remains underrepresented in the MIH literature.

The limitations should also be acknowledged. The sample size was modest, particularly for subgroup analyses and less frequent anomalies. The clinic-based convenience sample and single-center design may limit generalizability. In addition, the predominance of Hispanic participants and the grouping of all non-Hispanic participants into a single category limited evaluation of differences among specific racial or ethnic groups and may restrict generalizability to the broader, ethnically diverse U.S. pediatric population. Although MIH and non-MIH participants were drawn from the same clinical source population and DDA status was not used as a recruitment criterion, the convenience sampling approach may still have introduced selection bias. Children with more complex dental needs, including those with more severe MIH or other developmental findings, may have been more likely to attend a tertiary pediatric dental clinic or to have panoramic radiographs available. Therefore, the observed frequencies and strength of association may not be fully representative of the general pediatric population. Some sociodemographic variables had missing data. Dental caries was not included as a covariate in the present analysis. Accordingly, residual confounding related to caries experience cannot be excluded and should be considered when interpreting the observed associations. Panoramic radiographs have inherent limitations for the assessment of certain dental findings. Assessment of SRA on panoramic radiographs is subject to limitations related to magnification, projection geometry, patient positioning, and stage of root development. These factors may affect apparent crown-to-root proportions and should be considered when interpreting the SRA findings. Although the radiographic examiner was blinded to clinical MIH status and group assignment, radiographically visible changes in severely affected first permanent molars may have provided indirect clues to MIH status in some participants, and complete masking cannot therefore be assured. Although quantitative criteria were used for both SRA and taurodontism, these assessments were based on panoramic radiographs and may therefore be affected by projection geometry, patient positioning, and stage of root development. In addition, the overall association between MIH and DDAs was influenced substantially by SRA and taurodontism, and these findings should therefore be interpreted cautiously. Ectopic eruption may result from developmental, local, or mechanical factors, and the radiographic assessment used in this study did not permit determination of the underlying etiology. Finally, because of the cross-sectional design, the present results should be interpreted as evidence of association rather than causation. Larger multicenter studies with standardized protocols and longitudinal follow-up will be needed to confirm these findings and to clarify whether specific DDAs cluster with MIH in population-specific patterns.

## Conclusions

Within the limitations of this study, MIH was associated with a higher frequency of DDAs in this clinic-based cohort. This association remained significant after adjustment for country of birth, sex, and ethnicity. DDAs were numerically more frequent among children with moderate/severe MIH than among those with mild MIH, although this difference was not statistically significant. However, the observed association was influenced substantially by SRA and taurodontism, both assessed radiographically, and these findings should therefore be interpreted cautiously. Larger studies are needed to confirm these associations.

## Data Availability

The datasets generated and/or analyzed during the current study are not publicly available because they contain information that could compromise participant privacy, but are available from the corresponding author on reasonable request and subject to institutional and ethical approvals.
